# Quantitative Characteristics of Gene Regulation by Small RNA

**DOI:** 10.1371/journal.pbio.0050229

**Published:** 2007-08-21

**Authors:** Erel Levine, Zhongge Zhang, Thomas Kuhlman, Terence Hwa

**Affiliations:** 1 Center for Theoretical Biological Physics, University of California San Diego, La Jolla, California, United States of America; 2 Division of Biological Sciences, University of California San Diego, La Jolla, California, United States of America; Institute for Information Transmission Problems, Russian Federation

## Abstract

An increasing number of small RNAs (sRNAs) have been shown to regulate critical pathways in prokaryotes and eukaryotes. In bacteria, regulation by *trans*-encoded sRNAs is predominantly found in the coordination of intricate stress responses. The mechanisms by which sRNAs modulate expression of its targets are diverse. In common to most is the possibility that interference with the translation of mRNA targets may also alter the abundance of functional sRNAs. Aiming to understand the unique role played by sRNAs in gene regulation, we studied examples from two distinct classes of bacterial sRNAs in Escherichia coli using a quantitative approach combining experiment and theory. Our results demonstrate that sRNA provides a novel mode of gene regulation, with characteristics distinct from those of protein-mediated gene regulation. These include a threshold-linear response with a tunable threshold, a robust noise resistance characteristic, and a built-in capability for hierarchical cross-talk. Knowledge of these special features of sRNA-mediated regulation may be crucial toward understanding the subtle functions that sRNAs can play in coordinating various stress-relief pathways. Our results may also help guide the design of synthetic genetic circuits that have properties difficult to attain with protein regulators alone.

## Author Summary

The activation of stress response programs, while crucial for the survival of a bacterial cell under stressful conditions, is costly in terms of energy and substrates and risky to the normal functions of the cell. Stress response is therefore tightly regulated. A recently discovered layer of regulation involves small RNA molecules, which bind the mRNA transcripts of their targets, inhibit their translation, and promote their cleavage. To understand the role that small RNA plays in regulation, we have studied the quantitative aspects of small RNA regulation by integrating mathematical modeling and quantitative experiments in Escherichia coli. We have demonstrated that small RNAs can tightly repress their target genes when their synthesis rate is smaller than some threshold, but have little or no effect when the synthesis rate is much larger than that threshold. Importantly, the threshold level is set by the synthesis rate of the small RNA itself and can be dynamically tuned. The effect of biochemical properties—such as the binding affinity of the two RNA molecules, which can only be altered on evolutionary time scales—is limited to setting a hierarchical order among different targets of a small RNA, facilitating in principle a global coordination of stress response.

## Abstract 3

In bacteria, small RNAs can regulate the expression of genes at the translational level. The many advantages of this type of control include a tuneable threshold response and resistance to biochemical noise.

## Introduction

Small noncoding RNAs (sRNAs) have been demonstrated in recent years to play central regulatory roles in prokaryotes and eukaryotes [1–4]. Organisms that use sRNAs in post-transcriptional regulation range from bacteria to mammals. Interestingly, sRNAs are predominantly implicated in regulating critical pathways, such as stress responses in bacteria [5–15], or developmental timing and cell differentiation in plants and metazoans [16,17]. Despite the recent surge of interest in sRNAs, their regulatory role in bacteria has actually been a subject of research for the last several decades. Early on, sRNAs were mainly recognized for their specialized roles in controlling the transposition of insertion elements [18,19], in regulating plasmid copy number during plasmid replication [20–23], and in mediating plasmid maintenance through the toxin-antidote system [24]. Those sRNAs studied are encoded on the antisense strand and in *cis* with their targets [23,25], to which they bind through perfect base-pairing. This class of sRNAs will be referred to hereafter as antisense RNAs. In accord with their biological functions [25], some of these antisense RNAs are metabolically stable (e.g., the ones controlling transposition [26]), whereas others are very unstable (such as the ones controlling plasmid copy number [27,28]). For the latter, it has been demonstrated that the strength of inhibition is strongly related to the binding rate, rather than the binding affinity, of the antisense RNA and its target [29,30].

Until recently, only a few cases involving regulation by *trans*-encoded sRNA were known [31,32]. The advent of large-scale experimental techniques [33–36] and bioinformatic methods [35,37–39] has led to the identification and the subsequent verification of numerous such sRNAs in a variety of bacterial species in the past five years. Currently, there are over 70 such sRNAs identified in Escherichia coli [6,8,40]. Like regulatory proteins, these sRNAs can regulate the expression of multiple target genes, and are themselves regulated by one or more transcription factors. They have been implicated in the regulation of important pathways including oxidative response [15], osmotic response [13,32], acid response [9,10], quorum sensing [7], SOS response to DNA damage [11], glucose-phosphate stress[14], and more [5,6,8].

The mechanisms by which *trans*-acting sRNAs exert their effect are diverse. Most act by binding to the 5′ untranslated region (UTR) of a target mRNA [2,3,6], with specificity achieved through (often imperfect) base-pairing between the two RNA molecules. Upon binding, these sRNAs can reduce the efficiency of translation initiation—e.g., by interfering with ribosomal binding—or the stability of the target mRNA. Among these sRNAs that down-regulate their targets are RyhB (regulator of iron metabolism) [41–44], OxyS (oxidative stress) [15], and MicC and MicF (osmotic stress) [13,32]. In contrast, RprA and DsrA promote translation of their target, rpoS (encoding the stationary phase sigma factor σ^s^) [5], whereas GadY— the only sRNA in E. coli known to bind the 3′-UTR of its target— stabilizes its target [10].

A large class of *trans*-acting sRNAs bind tightly to the RNA chaperone Hfq, a highly abundant protein that also binds the target mRNA in a number of cases studied [15,45–48]. Binding to Hfq may protect these sRNA molecules from degradation in the absence of their mRNA targets [42,49–51]. Hfq has also been shown to facilitate the pairing of an sRNA with its target mRNA [43,52], leading to the inhibition of translational initiation. In turn, pairing of the sRNA and mRNA exposes both molecules to rapid degradation [42,43,49,53]. Importantly, the interaction between the sRNA and its target is noncatalytic in nature, since a given sRNA molecule may be degraded along with its target, instead of being used to regulate other targets [42].

Some antisense RNAs can also interact with their targets in a noncatalytic fashion. For example, the antisense RNA RNA-OUT forms a highly stable complex with its target RNA-IN, encoding the IS10 transposase [54]. With a half-life of over 2 h for this complex [55,56], the active antisense RNA may be regarded as irreversibly “consumed” by its target once the two bind. A similar stability is shown by CopA and its mRNA target [57], which codes for the R1 plasmid replication initiation protein RepA [28]. Although the extended base-pairing between the antisense RNA and its target eventually exposes the sRNA–mRNA complex to degradation by RNase III, this coupled degradation has little effect on repression itself [56,58]. Thus, for this class of sRNA regulators, repression is implemented by the irreversible sRNA–target complex formation, which is also noncatalytic.

The noncatalytic nature of sRNA–target interaction is qualitatively different from the catalytic effect of many protein regulators on the expression of their targets (e.g., protein regulators are not consumed upon regulating their targets). It is then interesting to ask whether sRNA-mediated regulation has special features distinct from protein-mediated regulation. Here we address this question using a combination of experimental and theoretical approaches. First, we describe the results of theoretical analysis that predicts a number of novel features for noncatalytic gene regulation by sRNAs. These features include a tunable threshold-linear expression pattern, a robust noise resistance characteristic, and a built-in capability for hierarchical cross-talk. These predictions are validated by a series of detailed experiments that quantified the regulatory effects exerted by the *trans*-acting sRNA, RyhB, on several targets in E. coli. We further extended the experiments to characterize the regulatory effect of the antisense RNA, RNA-OUT, to test the prediction that the novel features described above depended only on the noncatalytic nature of gene regulation and not necessarily on the degradation of the regulators themselves.

## Results

### Theoretical Analysis of the Noncatalytic Mode of Gene Regulation

**Figure 1:**
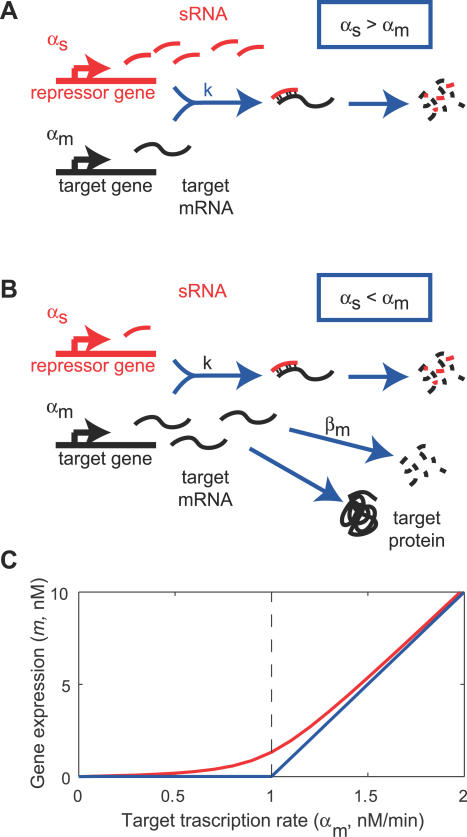
Threshold-Linear Response of a Target Gene (A) and (B) depict an idealized model for the interaction between mRNAs of a target gene and sRNAs. If the sRNA transcription rate is larger than that of the target (A), then gene expression is silenced, whereas if sRNA is transcribed less efficiently than its target (B), the residual unbound mRNAs code for proteins. (C) Predicted response curve of a target gene. The blue line depicts the idealized threshold-linear mode of regulation in which gene expression is completely silenced if the target transcription rate is below a threshold set by the transcription rate of the sRNA (indicated by the dashed line). Above this threshold, gene expression increases linearly with the difference between the mRNA and sRNA transcription rates. The idealized scenario is expected when binding between sRNA and mRNA occurs extremely rapidly. The red line is the actual response expected according to Equation 2, using the estimated parameters of Table 1, column 3 for α_s_ = 1 nM/min.

The noncatalytic nature of sRNA-mediated gene regulation suggests a novel threshold-linear mode of action, by which the expression of a target gene is silenced below a threshold, and is gradually activated above it (Figure 1). Consider first a case where sRNA and mRNA are co-degraded in a one-to-one fashion. In this case, if the transcription rate for the target mRNA (α_m_) is below that for the sRNA (α_s_) (Figure 1A), then most of the targets are expected to pair with the sRNAs and be rapidly degraded, as suggested recently by Lenz et al. [7]. Conversely, if the transcription rate of the mRNA exceeds that of the sRNA (Figure 1B), then most of the sRNAs are expected to turnover, whereas the unconsumed mRNAs are free to be translated into proteins. In the latter regime, the expressed protein level would reflect the difference between the two transcription rates. This scenario is summarized by the blue line in Figure 1C, where the steady state mRNA level of the target gene *(m)* is plotted against its transcription rate (α_m_). Messenger RNAs are expected to accumulate only if the target transcription rate exceeds the threshold, which is given by the transcription rate of the sRNA α_s_ (vertical dashed line).

**Table 1:**
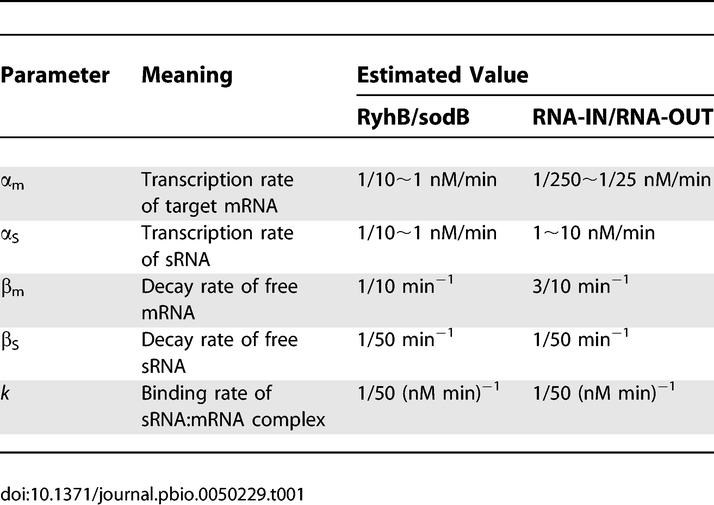
Model Parameters: Definitions and Estimated Values

The above qualitative prediction can be formulated quantitatively via a simple kinetic model for sRNA-mediated gene silencing. The model is cast in terms of two mass-action equations [7,59] for the cellular concentrations of the sRNA *(s)* and its target mRNA *(m)*

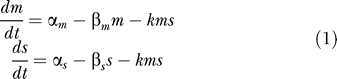



In this model, transcription of mRNAs and sRNAs are characterized by the rates α_m_ and α_s_, and their turnover by rates β_m_ and β_s_ respectively. The coupled degradation between sRNA and its target is described through a second-order kinetic constant *k*. The levels of Hfq and any endoribonuclease involved are assumed to be at saturation and are not tracked explicitly.

The predicted pattern of gene expression is obtained by solving Equation 1 in the steady state, with the steady state mRNA level

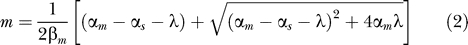
expressed in terms of the two control variables, α_m_ and α_s_, and an effective parameter λ = β_m_β_s_/*k*. The latter, being the ratio of the spontaneous and mutual turnover rates, characterizes the rate of mRNA turnover that is not due to the sRNA, and is referred to below as the leakage rate; it is a (inverse) measure of the strength of sRNA–mRNA interaction. For strong, rapid sRNA–mRNA interactions, the leakage rate is small and the solution (Equation 2) is given approximately by

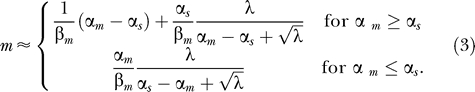



In the absence of leakage (i.e., λ = 0), Equation 3 is just the threshold-linear function depicted by the blue line of Figure 1C. For small but finite λ, the mRNA level is somewhat larger, especially near the threshold (where the denominators of the λ terms become small). Thus, leakage makes the transition smoother, as illustrated by the red line of Figure 1C, but does not change the qualitative feature of the threshold-linear form. We note that the value of the threshold (α_s_) is set by the sRNA transcription rate and is hence a dynamic variable that is controllable by the genetic circuit (rather than a fixed quantity such as the binding affinity encoded by the genomic sequence.) In particular, the threshold value is not affected by the strength of the interaction parameter *k* (as long as the leakage λ is reasonably small to preserve the threshold-linear form).

More generally, it is possible that degradation of the mRNA in the complex does not always lead to the degradation of the sRNA. Suppose that a fraction *p* < 1 of the sRNA is co-degraded with the mRNA. By repeating the above analysis, we find the same results, except that α_s_ and λ in Equations 2 and 3 are replaced by α_s_/*p* and λ/*p* (Text S1). Thus, partial co-degradation of the sRNA would effectively increase the threshold target transcription rate and also increase the leakage. However, it is not expected to change the form of the threshold-linear response. Alternatively, the effect of *p* < 1 could be accounted for by rescaling both axis of Figure 1C (i.e., *m* and α_m_) by a factor *p*. In a typical experiment, only the relative magnitudes of *m* and α_m_ are determined (see, e.g., Materials and Methods). Therefore, the value of *p* does not make a difference when confronting the predictions of the model with experimental data, and we will use the steady-state solution (Equation 2 or 3) below, regardless of the value of *p*.

The kinetic model (Equation 1) provides quantitative predictions given the knowledge of the different kinetic parameters. Below, we will apply the model to the sRNA RyhB [41–43,60], which is one of the best characterized *trans*-acting sRNAs and for which ample kinetic data exist for us to infer realistic values for all of the essential model parameters (see Materials and Methods, with the results summarized in Table 1). From these parameter values, we estimate a leakage rate λ ≈ 0.1 nM/min for RyhB. This is small but non-negligible compared with the relevant range of the transcription rates, α_m_ and α_s_. In fact, the smoothened threshold-linear expression pattern plotted in red in Figure 1C is the steady-state solution (Equation 2), with the RyhB parameters listed in the 3rd column of Table 1.

### Quantitative Experimental Characterization of RyhB

To validate the kinetic model (Equation 1), we tested experimentally its direct prediction, namely a smoothened threshold-linear response function as depicted in Figure 1C (red line). To this end, we characterized quantitatively the response of a target gene regulated by the sRNA RyhB. RyhB is involved in regulation of iron homeostasis in *E. coli,* and is expressed at low cellular iron levels. Its targets include iron-storage and oxidative response genes [41,60] whose expressions are needed to combat problems associated with elevated iron levels, but not when iron is deficient [61].

To circumvent the complex regulation of the endogenous system, we constructed a synthetic target gene, consisting of the 5′ control region and the first 11 codons of *sodB* (which is the strongest known natural target of RyhB [42,60]), translationally fused to the coding sequence of the reporter *gfp*. The target gene, *crsodB-gfp,* was driven by an inducible *lac* promoter, P_Llac-O1_ [62], and placed on the multi-copy plasmid pZE12S (see Materials and Methods). This construct allowed us to control the transcription rate of the target, α_m_, by changing the concentration of the inducer isopropyl β-d-1-thiogalactopyranoside (IPTG) in the medium. To quantify the relation between IPTG concentration and target transcription rate, we first characterized the bare target expression by transforming the pZE12S plasmid into a *ryhB^−^* strain of E. coli BW-RI. Expression of the target was assayed by measuring green fluorescent protein (GFP) fluorescence in the resulting cells grown in minimal M63 glucose medium with various amounts (0–0.5 mM) of IPTG (Figure S2). For each concentration of IPTG, we use the slope of the fluorescence versus optical density at 600 nm (OD_600_) plot (for OD_600_ < 0.2) to define the promoter activity (see Materials and Methods for a detailed description).

**Figure 2:**
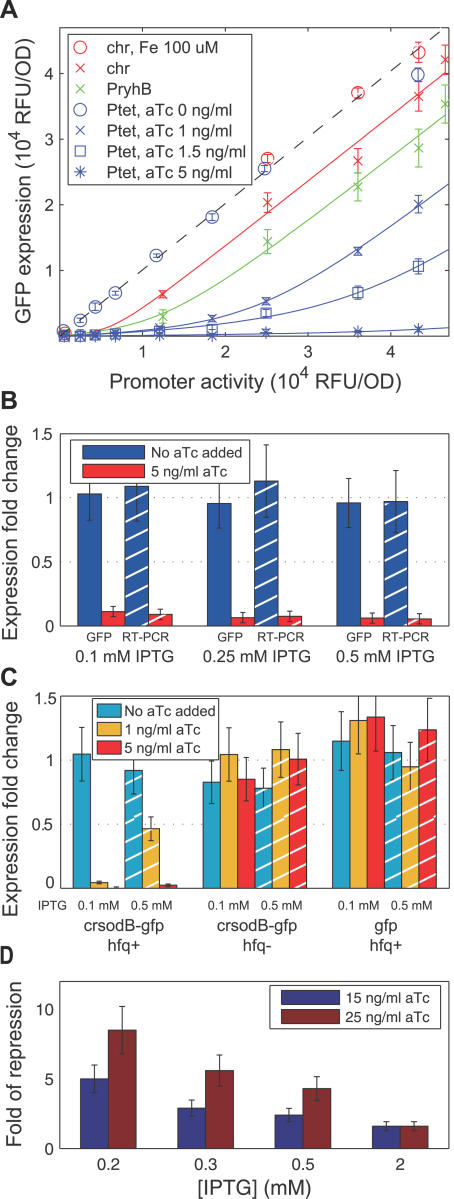
Threshold-Linear Response of a Reporter Target of RyhB (A) GFP expressions of various *rhyB^+^* strains (red: strain ZZS22, green: ZZS24, blue: ZZS23) are plotted against the promoter activity, defined as the GFP expression of the *ryhB* ^−^ strain (ZZS21) grown in identical medium (see Table 2 for information on the strains). Different promoter activities were obtained by varying IPTG concentration in the media (for example, the blue symbols were measured at 0, 0.05, 0.15, 0.2, 0.25 0.3, 0.4, 0.5 mM IPTG; see Figure S3). The curves are obtained from a single parameter fit of the data to the steady-state solution (Equation 2), as explained in the text and Table S1. (B) Ratio of GFP expression in the *ryhB^+^* strain ZZS23 (harboring P_Ltet-O1_:*ryhB*) and the RyhB-less strain ZZS21 measured through GFP fluorescence (solid bars) and RT-PCR (striped bars), for two different levels of aTc (blue and red) and three different levels of IPTG. In each case examined, the fold-change in GFP expression corresponded well to the fold-change in mRNA level. (C) Different RyhB levels (synthesized from P_Ltet-O1_ *:ryhB* driven by different levels of aTc) do not significantly change GFP expression in *hfq* ^−^ strains (middle group), or when crsodB-gfp is replaced by a *gfp* with a short 5′-UTR (right group). GFP fluorescence was measured in *hfq* ^−^ strains that express a plasmid-borne target (P_Llac-O1_:*crsodB-gfp*) with (ZZS23q) or without (ZZS21q) plasmid-borne RyhB. The ratio between the two at different levels of inducers is plotted in the middle group of bars. Similarly, GFP fluorescence was measured in strains carrying the pZE12G plasmid, in which the *gfp* structure gene with a short 5′-UTR is placed immediately downstream of the promoter, with (ZZS13) or without (ZZS11) plasmid-borne RyhB. The ratio between the two is plotted in the right group of bars. For comparison, data for the isogenic *hfq^+^* strains with the *crsodB-gfp* reporter are taken from (A) and replotted in the same format as the left group. (D) The fluorescence levels of cells expressing GFP by P_Llac-O1_:*crsodB-gfp* inserted chromosomally at the *attP* site was measured by flow-cytometry for strains ZZS43 (no *ryhB*), ZZS41 (plasmid-borne P_Ltet-O1_:*ryhB*), and ZZS01 (which contained no *gfp* gene). The latter was used to quantify the background fluorescence level. The fold of repression (vertical axis), is defined as [fluorescence(ZZS41) − fluorescence (ZZS01)]/[fluorescence (ZZS43) − fluorescence (ZZS01)]. The data show that the repression effect of RyhB is reduced at higher levels of IPTG, corresponding to larger transcription rates of the target. For the ease of comparison, the GFP fluorescence data of (A) is replotted in the same manner in Figure S4, where similar behavior is seen. These results show that the nonlinear effect of RyhB is also exhibited by a chromosomally encoded target.

We then repeated these measurements in cells harboring pZE12S and either chromosomal or plasmid-encoded *ryhB.* The expression rate of RyhB, α_s_, was controlled by a variety of means as detailed below. The GFP expressions at each level of RyhB expression (extracted from plots similar to Figure S2 for each strain) were then plotted against the above-defined promoter activity at the corresponding IPTG levels (Figure 2A).

**Table 2:**
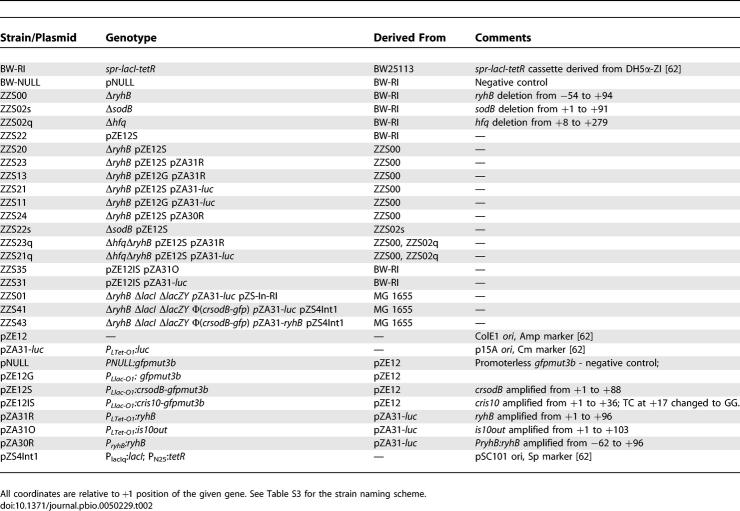
Bacterial Strains and Plasmids Used in This Study

As a calibration, we first observed that GFP expressions in wild-type *(ryhB* ^+^ *)* cells (strain ZZS22) grown in media with 100 μM FeSO_4_ (red circles) are indistinguishable from those of the *ryhB^−^* cells (strain ZZS21) (dashed black line), indicating the complete repression of RyhB activity at such a high iron level as expected. For the same wild-type (*ryhB* ^+^) cells grown in media with no added iron (red crosses in Figure 2A), GFP expressions were moderately reduced across all IPTG levels, and more so for a strain carrying a multi-copy plasmid that harbors *ryhB* driven by its native promoter (green crosses, strain ZZS24). These results are qualitatively consistent with the expected increase of RyhB expression upon reducing the iron level and upon adding multi-copy plasmid-borne sources.

To see the effect of even higher RyhB expression, we characterized GFP expression for a strain which was deleted of the chromosomal *ryhB* gene, but carried another plasmid harboring the *ryhB* structure gene driven by the strong synthetic P_Ltet-O1_ promoter [62] inducible by anhydrotetracycline (aTc) (strain ZZS23). In the absence of aTc in the growth medium (blue circles), GFP expression was essentially indistinguishable from that of the RyhB-less strain (ZZS21, dashed black line). The addition of small amounts of aTc (blue crosses, squares, and asterisks) drastically reduced GFP expression (up to 30-fold reduction compared with that of the RyhB-less strain). Altogether, using combination of chromosome and plasmid sources for RyhB, we present in Figure 2A the response of the target gene *crsodB-gfp* to varying promoter activities in the presence of six different levels of RyhB expression.

To verify that RyhB regulation was indeed achieved primarily through changes in the target mRNA level, we quantified the levels of the crsodB-gfp mRNA directly for strain ZZS23 (harboring plasmid-borne P_Ltet-O1_:*ryhB*) at two distinct levels of RyhB expression (corresponding to 0 and 5 ng/ml aTc added to the growth medium) and a variety of transcription levels for the *crsodB-gfp* target (0.1, 0.25, 0.5 mM IPTG in the growth medium) using quantitative real-time PCR (RT-PCR) (Figure 2B). We find that reduction in mRNA level is consistent with the corresponding reduction in GFP fluorescence; compare the solid and striped bars.

The interaction between RyhB and its endogenous targets depends on the RNA chaperone Hfq [41,43,49]. To demonstrate that the interaction between RyhB and the synthetic target, *crsodB-gfp,* shares this property, we repeated our measurements in strains deleted of *hfq*. In Figure 2C (middle group), we show the ratio between GFP fluorescence levels in a *hfq^−^* strain expressing RyhB from the P_Ltet-O1_ promoter (ZZS23q) and a RyhB-less *hfq^−^* strain (ZZS21q) for various levels of the inducers IPTG and aTc. We found that in the absence of *hfq*, the aTc dependence of the isogenic *hfq^+^* strain (Figure 2C, left group) was completely abolished, and GFP expressions all became the same as those of the RyhB-less strains (all of the bars of the middle group take on values ∼1). The results indicate that *hfq* is required for the repression effect observed here. This behavior is expected for a RyhB target, since RyhB accumulation and RyhB-target interaction requires Hfq [41–43,53,63].

As a different control, we characterized the GFP expression for *hfq^+^* strains in which the plasmid pZE12S was replaced by pZE12G, harboring the same P_Llac-O1_:*gfp* reporter, except that the 5′-UTR of the *gfp* gene was a short 27-base segment containing a strong ribosomal binding site instead of the *sodB* control region (see Materials and Methods). In Figure 2C (right group), we show the ratio between GFP fluorescence in a strain expressing RyhB from the P_Ltet-O1_ promoter (strain ZZS13) to that in the RyhB-less strain (ZZS11). We find that different degrees of RyhB expression have no effect on the observed GFP activity for all the inducer levels tested, indicating that the *sodB* control region is required for interaction.

### Reporter Target of RyhB Exhibits Threshold-Linear Expression Pattern

The data of Figure 2A reveal a spectrum of gene expression patterns: with RyhB expression strongly repressed (blue and red circle, red crosses), expression of the target gene was mainly controlled by the activity of its own promoter (controlled by IPTG), whereas for high RyhB expression (blue asterisks), target expression was greatly reduced regardless of the promoter activity. This qualitative behavior is what would be expected based on the model depicted in Figure 1. To make quantitative comparison with the predictions of the kinetic model (Equation 1), the data in Figure 2A were fitted to the steady-state solution of the model, Equation 2. This fit requires a single global parameter (associated with the leakage rate λ) and one additional free parameter (corresponding to the activity of the promoter expressing the sRNA, α_s_) per curve. The latter characterizes the position of the softened threshold, and is listed in Table S1 for each RyhB source studied. The corresponding best-fit curves are shown as the colored lines in Figure 2A.

The data of Figure 2A are fitted very well by softened threshold-linear form predicted by the model: a prominent feature of the predicted behavior—that target gene expressions all have the same linear dependence on its promoter activity at high expression levels much beyond the threshold, i.e., 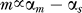 —is clearly reflected by the red, green, and the top blue curves for which the thresholds are much below the maximal promoter activity probed. The predicted threshold-linear response is best seen for intermediate RyhB expressions (green and blue crosses, blue squares); target expression was strongly repressed at low transcription levels, but turned up sharply for increasing activities of the target promoter.

Another way to present or view the threshold-linear response is that the fold-repression exhibited at a given RyhB transcription rate should decrease as the rate of target transcription increases. This is shown for the data of Figure 2A in Figure S4. We performed similar characterization for strains harboring a synthetic chromosomal target (P_Llac-O1_:*crsodB-gfp* inserted at the *attP* site); see caption of Figure 2D for details. While quantitative characterization of GFP expression becomes much more difficult for this chromosomally encoded target due to the low expression level, we see qualitatively from Figure 2D that the same trend is obtained.

### Threshold-Linear Response via Irreversible sRNA-Target Binding

As motivated in the theoretical study, we expect the threshold-linear response to be a generic feature of noncatalytic mode of gene regulation, not necessarily limited to sRNA-target pairs that undergo coupled degradation. For a number of the antisense RNA-target pairs, e.g., CopA/RepA of the R1 plasmid [57] and RNA-OUT/RNA-IN of the transposon IS10 [55,56], the pairing of the antisense RNAs with their respective targets was found to be irreversible but stable for hours. From the theoretical perspective, as long as the duplex does not dissociate back into the two active RNA components at relevant time scales, the system can still be described by the kinetic model (Equation 1) if we identify *m* and *s* as the free mRNA and sRNA concentrations. We thus expect the same smoothened threshold-linear response as described above.

We tested this prediction using RNA-OUT, the antisense sRNA that regulates the transposition of the IS10 insertion element in E. coli [18,19]. In IS10, the transposase gene (referred to here as *is10in*) is driven by the *pIN* promoter. Located only 35 bases downstream on the opposite strand is the *pOUT* promoter, which drives the transcription of the gene *is10out* encoding RNA-OUT. The prefect base pairing between the two RNA molecules at the 5′-UTR of *is10in* leads to a strong irreversible binding [55,56], which represses the translation of *is10in* [54] with only mild effect on its stability [56]. Quantitative data from previous experiments [26,56,64–68] in which RNA-OUT was expressed in both *cis* and *trans* allowed us to estimate key parameters for this sRNA-target pair (Text S1 and Table 1, column 4). Most of these parameters take on values similar to those we estimated for RyhB and its *sodB* target (Table 1, column 3). However, the degradation rate of RNA-IN, β_m_, is larger than the corresponding rate of typical RyhB targets [64], making the leakage rate λ larger. We therefore expect *is10in* to exhibit a somewhat smoother threshold-linear expression pattern. In the native IS10 system, however, the sRNA and its target are expressed in *cis*. This is likely to increase the sRNA-target binding rate *(k)* substantially, hence reducing the leakage λ and making the transition sharper.

To measure the effect of repression by RNA-OUT, we constructed a synthetic target consisting of a modified *is10in* control region translationally fused to *gfp.* The control region we use differs from that of the native *is10in* in two nucleotide positions, making its ribosome binding site (RBS) stronger (see [64] and Materials and Methods). The target gene, referred to as *cris10-gfp,* was inserted immediately downstream of the P_Llac-O1_ promoter in plasmid pZE12IS. Promoter activities at eight levels of IPTG (0–0.75 mM) were established as described before (Figure S2), by measuring GFP fluorescence in a strain (ZZS31) which carries pZE12IS but no RNA-OUT.

**Figure 3:**
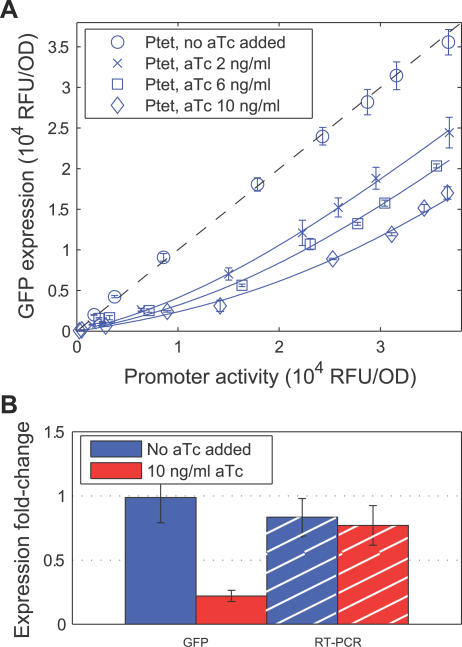
Threshold-Linear Response of a Reporter Target of RNA-OUT (A) GFP expressions of strain ZZS35 *(is10out^+^)* are plotted on the vertical axis against the promoter activity, defined (as in Figure 2) as the GFP expression of strain ZZS31 *(is10out^−^)* grown in identical medium. The different symbols correspond to the different levels of RNA-OUT expressed by the P_Ltet-O1_ promoter. The latter was controlled by varying amounts of aTc added to the growth medium (see legend). The solid lines are the steady-state solution (Equation 2) using the best-fit parameters listed in Table S2. (B) Ratio of GFP expression in *is10out+* (ZZS35) and *is10out^−^* (ZZS31) strains measured through GFP fluorescence (solid bars) and RT-PCR (striped bars). Expression of RNA-OUT was induced by 10 ng/ml aTc (red), whereas target expression was induced with 0.3 mM IPTG [corresponding to the 4th diamond from the right in (A)]. Change in the mRNA level of *cris10-gfp* (striped bars) is insignificant compared with changes in GFP fluorescence (solid bars).

As a controlled source of RNA-OUT, we used the pZA31O plasmid, which harbors the *is10out* gene driven by the strong synthetic P_Ltet-O1_ promoter [62]. We measured the response function at four different expression levels of RNA-OUT using different concentrations of the inducer aTc (0, 2, 6, and 10 ng/ml). The data obtained (symbols in Figure 3A) were fitted to the steady-state solution (Equation 2) as described above; best-fit parameters are given in Table S2. The fitted curves are presented as the solid lines in Figure 3A. In the absence of aTc, *cris10-gfp* expression coincides with that of the corresponding strain with no RNA-OUT source (dashed black line). At higher levels of aTc, the threshold-linear response is recovered, displaying a smooth transition as expected.

To verify that RNA-OUT repressed the translation of *cris10-gfp* mRNA without significantly altering its accumulation, we quantified the mRNA concentration of *cris10-gfp* using RT-PCR. The result is shown in Figure 3B. Whereas the GFP expression is repressed by more than 4-fold upon the addition of 10ng/ml aTc (solid blue and red bars), the mRNA levels were hardly affected by aTc addition (striped blue and red bars). Together, the results of Figure 3A and 3B validate the prediction that coupled degradation is not necessary for the threshold-linear form if the coupling between the sRNA and its target is irreversible.

### Hierarchical Cross-Talk between sRNA Targets

**Figure 4:**
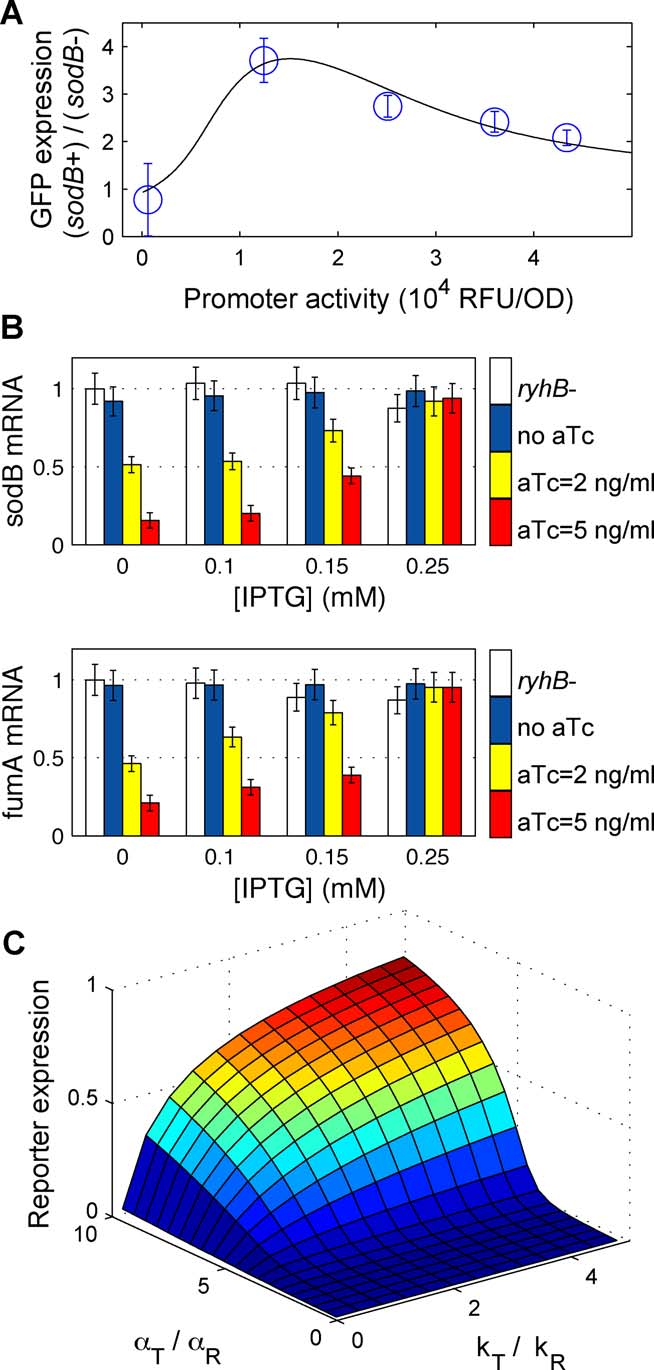
Cross-Talk between Different Targets of a Common sRNA (A) The fold-change between expression of the plasmid-borne reporter target *crsodB-gfp* in strain ZZS22 (*sodB^+^*) and strain ZZS22s (*sodB^−^*) cells. Promoter activity of the reporter was controlled by IPTG. The black line depicts the steady-state solution of a coupled-degradation model which is a straightforward generalization of the model (Equation 1) to the case of two targets (Text S1). (B) The effect of the expression of the multicopy target reporter on the expression of chromosomal targets. mRNA levels of two chromosomal RyhB targets, *sodB* and *fumA,* were determined using RT-PCR in strains with (ZZS23) and without (ZZS21) the *ryhB* plasmid. The repression effect of RyhB is measured as the ratio between mRNA levels in the two strains. As the expression level of the synthetic target, *crsodB-gfp*, is increased (by increasing IPTG concentration), the repression effect on the chromosomal targets is reduced. (C) Predictions of the coupled degradation model (Equation 5 of Text S1) for the expression of the reporter gene *(geneR)* to different transcription levels (α_T_) of another target *(geneT).* To generate the figure, we chose the transcription rate of *geneR* (α_R_) to be five times smaller than that of the sRNA, i.e., with α_S_ = 5α_R_. The ratio between the binding strengths of *geneT (k*_T_ *)* and *geneR (k*_R_ *)* to the sRNA determines the level of influence *geneT* has on the expression of *geneR,* and the abruptness at the onset of *geneR* activity. See text for details.

Some *trans*-acting sRNAs (including RyhB) have been shown to regulate multiple targets [6,8,60] whose expressions are independently regulated [69]. Because any one of the targets can reduce the level of sRNA, it is plausible for sRNA to mediate indirect interaction (cross-talk) between its different targets. In Figure 4A, we compare the expression of our reporter target, *crsodB-gfp,* in cells with and without the *sodB* structure gene (strains ZZS22 and ZZS22s respectively), both containing the chromosomal *ryhB* gene. Figure 4A shows that (i) the expression of the *crsodB-gfp* reporter is affected by the existence of the chromosomal *sodB* gene, with up to 4-fold higher expression in the *sodB* ^−^ mutant (strain ZZS22s), and (ii) the degree of enhanced reporter expression in this strain is dependent on the transcriptional activity of the reporter (the *x*-axis, controlled by IPTG). In comparison, no significant difference in expression was observed between *sodB^+^* and *sodB* ^−^ in *ryhB* ^−^ mutants (unpublished data). The results of Figure 4A suggest that the expression of the chromosomal *sodB* indeed interfered with the repression of *crsodB-gfp* by RyhB as anticipated.

It is straightforward to extend the kinetic model (Equation 1) to the case of multiple targets and account for the indirect interaction between them (Text S1). Assuming similar degradation rates for the two targets in the absence of the sRNA, the expressions for the mRNA level take the same functional form in the presence or absence of additional targets. To address the data of Figure 4A, we performed independent fits of the data of the *sodB^+^* strain (red line in Figure 2A) and the data of the *sodB* ^−^ strain. The ratio of the two is given by the black curve in Figure 4A. The shape of this curve shows that the effect of the *sodB* gene on the expression of the GFP reporter was peaked at a level of its promoter activity that corresponded to the expression threshold of the *sodB^+^* strain (ZZS22) (see the position of the kink of the red line of Figure 2A). This is a manifestation of the general prediction of the theory that target expression is most sensitive to changes in sRNA levels at the threshold, where the transcription of the sRNA and its target just balances.

Further evidences for cross-talk between different targets of RyhB are given in Figure 4B. The mRNA levels of two chromosomal RyhB targets (*sodB* and *fumA* [41,60]), as quantified by RT-PCR, are shown for different expression levels of the synthetic target gene *(crsodB-gfp)* driven by the P_Llac-O1_ promoter carried on the pZE12S plasmid. Open bars correspond to the control strain with no RyhB source (ZZS21), and colored bars correspond to different degrees of RyhB expression corresponding to different levels of aTc (in strain ZZS23). The *x*-axis indicates different levels of target expression, induced by IPTG. At basal expression level (no IPTG added), expression of the chromosomal targets is repressed by RyhB up to 10-fold (compare the blue and red bars for [IPTG] = 0). High expression of the plasmid target effectively rescues the chromosomal targets from repression ([IPTG] = 0.5 mM).

These data suggest that the cross-talk between different targets may allow for one target to relieve sRNA-mediated repression of another target. To explore this possibility, we used our model (Equation 5 in Text S1) to calculate the expression level of a reporter target *(geneR)* that is regulated by the same sRNA as another target gene *(geneT)*. We denote the transcription rates of the two genes by α_R_ and α_T_, respectively, and their binding rates to the sRNA by *k*_R_ and *k*_T_. The predicted dependence of *geneR* mRNA level on the ratio between transcription rates of the two genes (α_T_/α_R_ ), and the ratio between the two binding constants (*k*_T_/*k*_R_), is displayed in Figure 4C (where *geneR* mRNA level is measured in units of its level in the absence of the sRNA). In this figure, transcription rate of *geneR* is chosen to be 5 times smaller than that of the sRNA. Therefore, in the absence of *geneT (*α_T_ = 0), expression of *geneR* is strongly suppressed by the sRNA.

Figure 4C portrays a hierarchical cross-talk effect: the expression of a weakly interacting target (e.g., *geneR,* with a small *k*_R_) is highly affected by another target that is more strongly interacting (e.g., *geneT,* with *k*_T_ > *k*_R_); see the large *k*_T_/*k*_R_ region of Figure 4C, where the expression of *geneT* (increasing α_T_/α_R_) indirectly activates *geneR* by relieving the sRNA repression. Conversely, a strongly interacting target (e.g., *geneR,* with a large *k*_R_) is expected to be much less affected by a weakly interacting one (e.g., *geneT,* with *k*_T_ < *k*_R_). Thus, in the small *k*_T_/*k*_R_ region of Figure 4C, the expression of *geneR* remains suppressed even when *geneT* is highly expressed. Interestingly, our calculation predicts that for large *k*_T_/*k*_R_, the response of *geneR* to changes in the transcription rate of *geneT* may be very sharp. For example, the data of Figure 4C allow for an effective Hill coefficient ∼10 for *k*_T_/*k*_R_ ≈ 2. Thus, the sensitivity of the sRNA-mediated repression may be translated into sensitivity in the indirect interaction between its targets.

## Discussion

The “standard model” of gene regulation in bacteria primarily involves transcriptional initiation control by one or more regulatory proteins. Solid understanding of the key mechanistic ingredients of transcriptional regulation [70], stemmed from decades of research in molecular biology, leads to a reasonable quantitative description [71–73]. Although such a framework for sRNA is still lacking, the successful description of our experimental results by the simple kinetic model (Equation 1) for sRNA-mediated regulation prompted us to use this model to compare between the two modes of regulation.

### sRNA Regulation Is Subject to Dynamic Control

**Figure 5:**
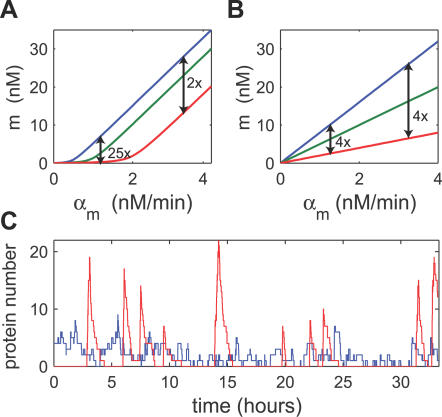
Comparison between sRNA- and Protein-Mediated Repression (A) Steady-state solution of model (1), with the estimated parameters of Table 1. The strength of sRNA repression decreases as the target transcription increases. (B) Steady-state solution of a model for protein regulators (Supporting Text S1), where the strength of repression is independent of target transcription rate. (C) Temporal behavior in a single stochastic simulation [94] of the expression of two model genes, *geneA* (blue line) and *geneP* (red), regulated by sRNA and protein regulators respectively. For *geneA* we set α_*A*_ = 1/min and *k_A_* = 0.02/min, while for *geneP* we have *α_P_* = 0.0043/min and *k_P_* = 0. All other parameters are taken from Table 1 and are identical for both genes. This choice of parameters makes the mean mRNA levels of the two genes equal. The bursty nature of the noise for *geneP* is compared with the smooth fluctuations exhibited by *geneA*.

Analysis of a simple model of protein-mediated gene regulation (Text S1) predicts that regardless of whether a protein regulator acts as a transcriptional repressor or as a catalyst of mRNA degradation, target expression always increases linearly with the promoter activity. The ratio between expression levels at different concentrations of the regulator is independent of the target activity (Figure 5B). Thus, one can safely talk about the strength of repression in term of the fold-change in gene expression in the presence and absence of the repressor without referring to the rate of target transcription.

This is, however, not the case for the threshold-linear mode that characterizes sRNA-mediated regulation. Here the fold-change depends not only on the presence of the repressor, but also on the transcription of the target (Figure 5A, arrows). For the same degree of repressor transcription (e.g., compare the red and blue lines), the fold repression could be small (e.g., 2-fold) above the threshold and large (e.g., 25-fold) below the threshold. This property may have functional consequences: sRNA may serve to tightly shutdown a gene that is repressed by other means. However, at circumstances that allow for high expression of the target, sRNA expression may exert virtually no effect. Moreover, in the threshold-linear mode of sRNA-mediated gene regulation, the onset of repression is set by comparison of transcription rates between sRNA and its target. As a result, the threshold value is dynamically tunable through controlling the rate of sRNA transcription. In contrast, protein–operator binding affinity, which controls the onset of repression in protein-mediated regulation, is fixed genetically by the operator sequence. Dynamic control of the latter would require other cofactor(s) and auxiliary binding sites and become more elaborate to implement. Of course, the more complex mode of control described here for sRNA can, in principle, be realized through more complex promoters involving more complex protein–protein interactions [74]. Also, features of sRNA-mediated regulation discussed here may also be realized by proteins that regulate the proteolysis of their targets in noncatalytic ways. In the latter case however, the steady co-degradation of protein regulators may pose a substantial metabolic load.

In a number of cases studied, a sRNA serves as a node in a regulatory cascade. Expression of the sRNA may be controlled by protein regulator that senses (directly or indirectly) an environmental signal. For example, the Ferric Uptake Regulator (Fur) is activated by free Fe^2+^ ions and negatively regulates transcription of RyhB, which in turn regulates targets whose expressions are required when Fe^2+^ is abundant in the cytoplasm [41,60]. Our results suggest that sRNA regulators may be more than a simple “inverter” of such a protein regulator. sRNA regulators could act, for example, as a “stress-relief valve.” In the iron example, whereas Fur senses levels of Fe^2+^ continuously (through rapid equilibration between Fur and Fur–Fe^2+^), we predict that targets of RyhB will only be expressed when the Fe^2+^ level crosses some threshold. This threshold can be set dynamically for each target by regulators controlling its transcription.

Recently it has been suggested that targets of microRNA regulation in eukaryotes may be classified as “switch,” “tuning,” and “neutral” targets, depending on their response to microRNA level [75,76]. In the framework presented here, these classes correspond to targets whose transcription rate is well below, near, or well above that of the RNA regulator. We emphasize, however, that the threshold-linear picture we draw is only applicable if the level of the free RNA regulator is affected by its interaction with its targets, i.e., for regulators that operate in the noncatalytic mode. This is yet to be established for microRNAs in eukaryotes.

### sRNAs May Exhibit Tight Repression of Fluctuations

Our model predicts that deep in the repressed state, the sRNAs strongly repress variations in protein expression. The effect of noise on gene expression is a subject of extensive current research [77–80]. We studied this effect theoretically by generalizing the model (Equation 1) to incorporate stochastic fluctuations (Text S1). In Figure 5C, we compare results of stochastic simulations for two genes with the same low mean protein expression: *geneA* is silenced by a sRNA, and *geneP* is repressed transcriptionally by a protein regulator. In general, we predict a much-reduced variance in protein level for sRNA-mediated regulation (Text S1). This can be understood by inspecting the time courses of protein expression (Figure 5C). With the protein regulator (red curve), any leakage in transcription is amplified through translation, resulting in large bursts of protein expression, as was recently observed experimentally [81,82]. With the sRNA (blue curve), gene expression is expected to be much smoother, because mRNA molecules are rarely translated. This difference in the noise properties may be very important in situations where a large burst of proteins will switch a cell from one stable state to another. In cases such as stress responses where unintentional entry into the alternative state may be harmful and spontaneous switching is to be avoided, sRNA-mediated regulation might possess a distinct advantage. Attenuation of noise by decreased burst size may also be accomplished by eukaryotic microRNAs [76], through a decrease in mRNA stability or inhibition of translation.

### sRNA Regulation May Be Highly Sensitive

sRNA-mediated regulation was predicted to be ultrasensitive to small changes in sRNA expression near the threshold [7]. A common measure for the abruptness of a transition, referred to as the “sensitivity,” is the maximal slope of the response curve, *m*(α_s_), in a double-log plot. From the solution (Equation 2), we find this sensitivity to be given by 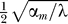 , which quantifies our statement that lower leakage makes a sharper transition, and also predicts a sharper transition for highly expressed targets. For sRNA regulators described by the parameters of Table 1, we find the sensitivity to be given approximately by 2.5 for α_m_ = 1 nM/min, and 4.3 for α_m_ = 3 nM/min . In comparison, the sensitivity of a protein repressor is bounded by the Hill coefficient, which is typically ≤2, although higher sensitivity (3∼4) can also be accomplished via, e.g., DNA looping [73]. On the other hand, much higher sensitivity can be achieved by processes such as those with zeroth-order kinetics [83].

### Hierarchical Cross-Talk between Targets of sRNA

Our data demonstrate how the activity of a strong target of RyhB may influence the expression of another target. In particular, we show that over-expression of a plasmid-borne target relieves completely the strong sRNA repression of its chromosomal target. Generalizing our kinetic model offers a simple intuitive picture (Figure S1). A weak sRNA target *(geneR)* is completely repressed by the sRNA when another, stronger target *(geneT,* with *k*_T_ ≫ *k*_R_ *)* is not expressed (Figure S1A). Expression of the latter captures a significant portion of the sRNAs, thus allowing some mRNA molecules of *geneR* to be translated into proteins (Figure S1B). On the other hand, expression of another target weaker than *geneR* may not attract enough sRNA to affect the expression of *geneR* (unpublished data).

In the context of a single target, our model predicts that the strength of the sRNA–target interaction influences only the smoothness of the transition, but not the threshold value of the threshold-linear expression pattern. However, when multiple targets are expressed simultaneously, the different mRNA species are expected to compete for association with the same pool of sRNA, and the relative interaction strength becomes a key determinant of the complex interactions that ensue. The interaction strength of the different targets sets their relative position in the cross-talk hierarchy, where targets of a given binding strength affect—but are not affected by—targets of lower binding strength.

Through quantitative characterization of gene regulation for two distinct classes of sRNA regulators, we have shown that sRNA-mediated regulation has many functional properties that are fundamentally different from the classical, protein-mediated mode of gene regulations. Analysis of our model suggests that sRNAs may offer tight regulation below the threshold (repressing the average expression and reducing fluctuations) accompanied by derepression away from the threshold. Taken together, this suggests that sRNAs working in the threshold-linear mode may be particularly suitable for a “stress-relief” mechanism, where no action is elicited until a tolerance threshold is exceeded. Knowledge of these properties is essential to an integrated understanding of gene regulatory systems, and may inspire the design and synthesis of novel genetic circuits [84] with properties difficult to obtain by using regulatory proteins alone.

## Materials and Methods

### Strains and plasmids.

All experiments were performed with BW-RI cells derived from E. coli K-12 BW25113 [85], with the transfer of the *sp^r^-lacI-tetR* cassette from DH5α-ZI cells [62] by phage P1 transduction. This cassette provides the constitutive expression of *lacI* and *tetR* genes [62]. For some experiments, *ryhB* and/or *sodB* were deleted from BW-RI [85]. These strains were then transformed by the following target and source plasmids. All strains and plasmids used are summarized in Table 2.

pZE12-*luc,* whose copy number has been estimated at 50–70 copies [62], was used to make the target plasmid pZE12S. Using site-directed mutagenesis, an *EcoR*I site was created by adding GAAT immediately downstream of +1 of the P_Llac-O1_ promoter. The region between the newly created *EcoR*I site and the resident *EcoR*I site 6 bp upstream of RBS was then deleted by *EcoR*I digestion and subsequent religation, yielding pZE12-*lucM*. The *Kpn*I-*Xba*I flanking *luc* gene in pZE12-*lucM* was replaced by the *gfpmut3b* structure gene [86]. This yields pZE12G, which harbors the P_Llac-O1_:*gfpmut3b* construct with a 5′-UTR defined by an *EcoR*I site immediately downstream of +1 and a *KpnI* site immediately upstream of the translation start of *gfpmut3b*. The 15-base sequence sandwiched by the *EcoR*I and *Kpn*I sites, ATTAAAGAGGAGAAA, contains an RBS indicated by the underlined bases. The 5′-UTR from the control region of *sodB* (*crsodB*, from −1 to +88 relative to the transcriptional start site of *sodB* and including the first 11 codons) was cloned into the *EcoR*I and *Kpn*I sites of pZE12G, yielding pZE12S. pZE12S therefore contains the ColE1 *ori,* the P_Llac-O1_ promoter [62], and *crsodB* fused to the coding sequence of the *gfpmut3b* gene. Similarly, the control region of *is10in* (from +1 to +36) was substituted for *crsodB* in pZE12S, yielding pZE12IS. To improve the expression level, the RBS in the *is10in* control region was modified by changing TC (+16 to +17) to GG.

Three sRNA-source plasmids (pZA30R, pZA31R, and pZA31O), were derived from the pZA31-*luc* plasmid, which has been estimated to maintain at 20–30 copies per cell [62]. Each contains the p15A replication *ori* and is marked by chloramphenicol resistance. First, a *Nde*I site was added immediately downstream the +1 of the *luc* gene by inserting ATG between +2 and +3, and a *BamH*I site was added downstream of *luc* by inserting ATC between the 1,772th and 1,773th nucleotides, yielding pZA31-*lucNB*. For pZA31R, the *ryhB* gene (from +1 to +96 cloned directly from E. coli K-12) was ligated into the *Nde*I/*BamH*I sites of pZA31-*lucNB*, replacing the *luc* gene. For pZA30R, the P_Ltet-O1_ promoter and the *luc* gene of pZA31-*lucNB* were replaced by P_ryhB_:*ryhB* (from −62 to +96 cloned directly from E. coli K-12 MG1655), which contains the *ryhB* gene and its native promoter. Finally, for pZA31O, the *is10out* gene (from +1 to +103) was substituted for the *luc* gene in pZA31-*lucNB*.

In addition, we transferred the target *crsodB-gfp* to the *attP* site of strain ZZS00 *(ryhB*−*)* chromosome using the method of Diederich et al. [87]. Briefly, a *Sal*I/*BamH*I-flanked P_Llac-O1_: *crsodB*-*gfpmut3b* containing the downstream terminator was cloned into the same sites of pLDR10 containing the attachment site *attP* and encoding the chloramphenicol (Cm) resistance. The recombinant plasmid was digested with *Not*I and the larger portions of the plasmids containing the fragment of interest but not the *ori* were religated. The circular DNA molecules were transformed into ZZS00 cells expressing the *int* gene contained in pLDR8, a helper plasmid bearing a temperature-sensitive *ori* and encoding the kanamycin (Km) resistance. The transformations were applied on LB+Ap plates that were incubated at 42 °C. The transformants were tested for sensitivity to Cm and Km. The ampicillin (Ap)-resistant but Cm- and Km-sensitive transformants were identified as the clones that carry the DNA fragment of interest at the *attP* site of E. coli chromosome.

### Medium, growth, measurements.

BW-RI strains each containing the target and/or source plasmids were grown in M63 minimal media with 0.5% glucose, and standard concentrations of the appropriate antibiotics. The overnight cultures were diluted into fresh M63 media (OD_600_ ≈ 0.002) containing the appropriate antibiotics as well as varying amounts of the inducers (aTc, IPTG, FeSO4) in the wells of 48-well plates. The plates were incubated with shaking at 37 °C and taken for OD_600_ and fluorescence measurements every hour for up to 12 h (until a final OD_600_ of 0.2–0.3) using a TECAN Genios-Pro plate reader (http://www.tecan.com). Each measurement was repeated 3–6 times and the data were analyzed as discussed below.

For RT-PCR measurements, overnight cultures were used to inoculate M63 medium with 0.5% glucose, standard concentrations of the appropriate antibiotics, and various concentrations of inducers to an initial OD_600_ of 0.001 and grown in 48-well plates in a 37 °C incubator. OD_600_ and GFP fluorescence were monitored periodically (if applicable). When OD_600_ of these cultures reached 0.3–0.5, approximately 10^9^ cells of each culture were harvested in a microcentrifuge at 4 °C, treated with 10 mg/ml lysozyme in TE buffer (pH = 8.0) and total RNA was collected using an Absolutely RNA miniprep kit (Stratagene; http://www.stratagene.com). The prepared samples were then treated with Turbo DNA-free DNase (Ambion; http://www.ambion.com), and PCR controls were performed on each sample to verify the absence of contaminating DNA. cDNA was prepared with 1 μg of RNA from each sample using Superscript III First Strand Synthesis system (Invitrogen; http://www.invitrogen.com). Dilutions of the resulting samples were then used as the template in PCR reactions using iQ SYBR Green Supermix (Bio-Rad; http://www.bio-rad.com) in a Smart Cycler thermal cycler (Cepheid; http://www.cepheid.com).

To measure expression from a chromosomal target, cells were grown overnight in minimal media with antibiotics. Cultures were then diluted to OD_600_ = 0.001, and grown in a 12-well plate with 3 ml of culture in each well, with appropriate antibiotics and inducers. To determine the growth rate, OD_600_ was measured every 60 min. Cultures were grown at 37 °C with constant shaking until they reach OD_600_ = 0.3, at which time 1.7 ml of each culture was spun down and resuspended in 1 ml phosphate buffer solution (PBS). GFP fluorescence was measured using a Becton-Dickinson FACSCalibur flow cytometer with a 488-nm argon excitation laser and a 515- to 545-nm emission filter (FL1) at a low flow rate. Photomultiplier tube (PMT) voltage was set to 950 V, and a linear amplifier was set at 9.5×. Forward scatter and fluorescence values were collected for 50,000 cells.

### Data analysis.

To obtain gene expression patterns for the different strains, we averaged (for each time point) the data obtained from the different repeats for each combination of strain and inducers. First, the cell doubling rate (μ) was obtained as the slope of a linear fit of log_2_(OD_600_) versus time for each strain and condition; this yielded a doubling time of ∼2 h for most strains. Next, for all of the time points concerning each strain and condition, we plotted the average fluorescence versus average OD_600_ on linear-linear plot and extracted the slope *(f)*. In Figure S2 we show, for example, GFP fluorescence against OD_600_ for the *ryhB* ^−^ strain (ZZS21), together with the fitted slopes. Each slope gave the average fluorescence per growing cell (in unit of relative fluorescence units (RFU)/OD) for that strain and the corresponding growth condition. The raw fluorescence production rate per cell was computed as fμ(1 + μτ) [88], upon taking into account of the maturation kinetics of GFPmut3 (maturation half-life τ of ∼30 min) [86]. We then subtracted away from this raw rate the background fluorescence production rate, obtained in the same way from data collected from our negative control strain BW-NULL. This yielded the rate of GFP production synthesis from P_Llac-O1_, and is referred to as the GFP expression. The results were plotted in Figure S3 at each IPTG level for different levels of RyhB expression, via the P_Ltet-O1_ promoter controlled by the amount of aTc in the growth medium.

To fit the experimental data with the steady-state solution (Equation 2), we assume that the GFP expression defined above is proportional to *m*, the steady-state mRNA level, i.e., GFP expression = *bm,* where *b* reflects the rate of GFP translation and maturation. Then, Equation 2 can be written in the following way,

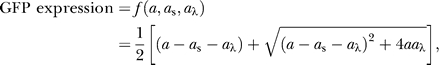
where 
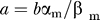
 is the GFP expression in the absence of the sRNA, referred to as the promoter activity and set by the IPTG concentration, 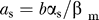 is proportional to the transcription rate of the sRNA (and therefore takes different values for different experiments); and 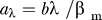 is proportional to the leakage parameter (defined in Results). The latter is independent of the sRNA activity, and should be chosen once for all experiments. We fitted the data to *f*(*a*,*a*_a_,*a*_λ_) using a standard Levenberg-Marquardt algorithm implemented in MATLAB (MathWorks; http://www.mathworks.com), with the least-square error defined as

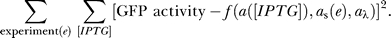



The values of the best-fit parameters obtained are given in Table S1 in terms of 0.5 confidence intervals.

### Estimation of model parameters.

The values of the model parameters can be estimated from various experiments. Consider first RyhB and its targets [41,42]. In the absence of its targets, the Hfq-bound sRNA RyhB is very stable, with a half-life of ∼30 min [42,49], yielding β_s_ ∼ 1/50 min^−1^. Similarly, from the half-life of ∼6 min for *sodB* mRNA [42] in the absence of RhyB, we have β_m_ ∼ 1/10 min^−1^ . Moreover, DNA microarray experiments [69,89] indicated approximately 10–20 copies/cell for the *sdhCDAB* and *sodB* mRNA in rich medium (where iron is abundant and RyhB is expected to be repressed). This suggests a target transcription rate (α_m_) of ∼ 1 nM/min in the state where mRNA is expressed. In general, α_m_ is controlled by various cellular signals (e.g., *sdhCDAB* by Crp-cAMP) and can typically vary ∼10-fold. (The DNA microarray study of Zhang et al. [69] showed approximately 5-fold change in *sdhCDAB* and *sodB* mRNA levels under various physiological conditions.) On the other hand, the activity of the RyhB promoter has a broad range, since it is strongly regulated by Fur-Fe^2+^ whose concentration can vary over 1000-fold [61]. We model the latter by allowing α_s_ to take on the range from 0.1/min to 10/min. Finally the coupled degradation rate *k* can also be deduced from the results of Masse et al. [42] (assuming *p* of order 1). Because RyhB is shown to disappear in the presence of its targets within 3 min, then by using an estimated target mRNA concentration of 20 nM, we find 1/50 (nM min)^ −1^, which is close to the diffusion-limited association rate for typical small proteins [90,91] and is similar to what has been observed directly for the sRNA OxyS and its target *fhlA* [92], as well as for the antisense *hok/sok* pair [93].

Finally, we consider RNA-OUT and its target, the mRNA of *is10in*. RNA-OUT itself is extremely stable, with a half-life dictated by dilution due to growth β_s_ ∼ 0.02 min^−1^ [26], while the half-life of *is10in* mRNA is typical to bacterial mRNA (2–3 min, β_m_ ∼ 0.3 [64]). Binding of RNA-OUT to its target mRNA is characterized by a second-order binding constant in the range of *k ∼* 1/50–1/20 (nM min)^ −1^. The *pOUT* promoter is a typical promoter, and we assume that α_s_ is not very different from that of RyhB [65]. The *pIN* promoter, on the other hand, is atypically weak, and is only enhanced 10-fold upon methylation [65–67].

Values of the model parameters are summarized in Table 1.

## Supporting Information

Figure S1 Model for Indirect Interaction between Different Targets of a sRNA, in the Case *k*_T_ ≫ *k*_R_
When *geneT* is not expressed, the sRNA silences the expression of *geneR*. When *geneT* is expressed, most sRNA molecules bind and degrade with mRNAs of *geneT,* allowing mRNAs of *geneR* to be translated into proteins.
(63 KB PDF)

Figure S2 Example for Raw Data, Used to Compile Figure 2A GFP fluorescence is plotted against OD_600_ for the RyhB-less strain (ZZS21) containing the plasmid borne P_Llac-O1_:*crsodB-gfp* reporter. Lines are given by a linear fit. The slope of each line was used to define the GFP expression.
(55 KB PDF)

Figure S3 Example for Raw Data, Used to Compile Figure 2A GFP expression for strains (ZZS23) harboring P_Ltet-O1_:*ryhB* on a plasmid, in addition to the P_Llac-O1_:*crsodB-gfp* reporter. The IPTG dependence of GFP expression (defined from plots such as Figure S2) is plotted for different degrees of RyhB expression. The latter is controlled by the level of the inducer aTc in the growth medium as indicated by the legend.
(46 KB PDF)

Figure S4 Repression Strength of RyhB Depends on the Transcription Rate of the Target The fluorescence levels of cells carrying a plasmid coding for the target, P_Llac−O1_:*crsodB-gfp,* was measured as in Figure 2A, for strains ZZS21 (no *ryhB*) and ZZS23 (plasmid-borne *ryhB*). The fold of repression (vertical axis) is defined as the ratio between the two. The repression effect of RyhB is diminished at higher levels of IPTG, corresponding to higher transcription rates of the target.
(45 KB PDF)

Table S1 Best-Fit Parameters of the Data in Figure 2A to Model (Equation 1), Given in Terms of 50% Confidence Interval See Material and Methods for a detailed description of the fitting procedure.
(13 KB PDF)

Table S2 Best-Fit Parameters of the Data in Figure 3A to Model (Equation 1), Given in Terms of 50% Confidence Interval See Material and Methods for a detailed description of the fitting procedure.
(13 KB PDF)

Table S3 Naming Scheme for Strains Used in this Study (13 KB PDF)

Text S1 Detailed Description of Models and Derivation of Analytical Results (120 KB PDF)

### Accession Numbers

The GenBank (http://www.ncbi.nlm.nih.gov/Genbank/) accession numbers for the genes and gene products discussed in this paper are *ryhB* (GeneID: 2847761), *sodB* (GeneID: 944953), *fumA* (GeneID: 2955664), *fur* (GeneID: 945295), *hfq* (GeneID: 948689), *oxyS* (GeneID: 2847701), *micC* (GeneID: 2847713), *micF* (GeneID: 2847742), *rprA* (GeneID: 2847671), *dsrA* (GeneID: 946470), *rpoS* (GeneID: 947210), *gadY* (GeneID: 2847729).

## Abbreviations

aTc: anhydrotetracycline
GFP: green fluorescent protein
IPTG: isopropyl β-d-1-thiogalactopyranoside
OD: optical density
sRNA: small RNA
UTR: untranslated region
