# Correction: Quantitative Characteristics of Gene Regulation by Small RNA

**DOI:** 10.1371/journal.pbio.0060005

**Published:** 2008-01-29

**Authors:** Erel Levine, Zhongge Zhang, Thomas Kuhlman, Terence Hwa

Correction for:

Levine E, Zhang Z, Kuhlman T, Hwa T (2007) Quantitative characteristics of gene regulation by small RNA. PLoS Biol 5(9): e229. doi:10.1371/journal.pbio.0050229

The authors would like to add the second author, Dr. Zhang, as a second corresponding author. He can be contacted by e-mail at zzhongge@biomail.ucsd.edu.

