# Supplementary material for: Quantitative Characteristics of Gene Regulation by Small RNA
Source: PLoS Biol. 2007 Aug 21;5(9):e229. doi: 10.1371/journal.pbio.0050229 (PMC1994261; doi:10.1371/journal.pbio.0050229)
Supplement: Figure S1 — When geneT is not expressed, the sRNA silences the expression of geneR. When geneT is expressed, most sRNA molecules bind and degrade with mRNAs of geneT, allowing mRNAs of geneR to be translated into proteins. (63 KB PDF) [file pbio.0050229.sg001.pdf]

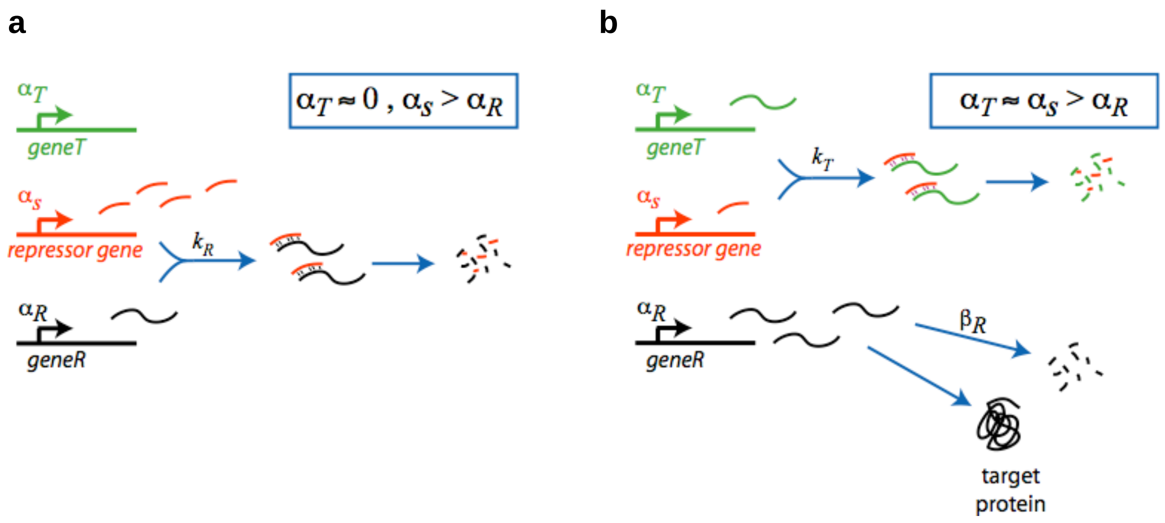

Figure S1. Model for indirect interaction between different targets of a small RNA, in the case $k_T \gg k_R$. When *geneT* is not expressed, the small RNA silences the expression of *geneR*. When *geneT* is expressed, most sRNA molecules bind and degrade with mRNAs of *geneT*, allowing mRNAs of *geneR* to be translated into proteins.
