# Supplementary material for: Quantitative Characteristics of Gene Regulation by Small RNA
Source: PLoS Biol. 2007 Aug 21;5(9):e229. doi: 10.1371/journal.pbio.0050229 (PMC1994261; doi:10.1371/journal.pbio.0050229)
Supplement: Figure S2 — GFP fluorescence is plotted against OD600 for the RyhB-less strain (ZZS21) containing the plasmid borne PLlac-O1:crsodB-gfp reporter. Lines are given by a linear fit. The slope of each line was used to define the GFP expression. (55 KB PDF) [file pbio.0050229.sg002.pdf]

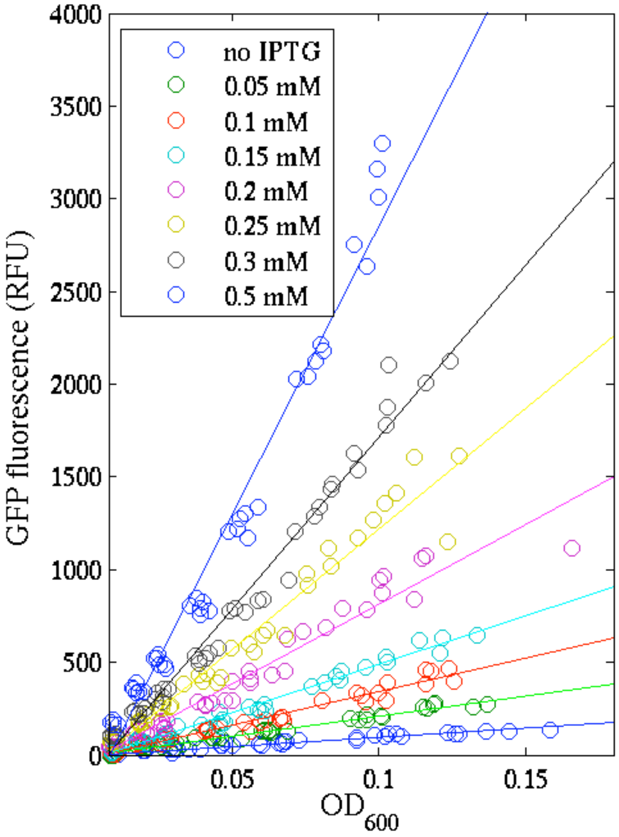

Figure S2. *Example for raw data, used to compile Fig. 2a of the main text*. GFP fluorescence is plotted against OD600 for the RyhB-less strain (ZZS21) containing the plasmid borne $P_{Llac\text{-}O1}$:*crsodB-gfp* reporter. Lines are given by a linear fit. The slope of each line was used to define the "GFP expression".
