# Supplementary material for: Quantitative Characteristics of Gene Regulation by Small RNA
Source: PLoS Biol. 2007 Aug 21;5(9):e229. doi: 10.1371/journal.pbio.0050229 (PMC1994261; doi:10.1371/journal.pbio.0050229)
Supplement: Figure S3 — GFP expression for strains (ZZS23) harboring PLtet-O1:ryhB on a plasmid, in addition to the PLlac-O1:crsodB-gfp reporter. The IPTG dependence of GFP expression (defined from plots such as Figure S2) is plotted for different degrees of RyhB expression. The latter is controlled by the level of the inducer aTc in the growth medium as indicated by the legend. (46 KB PDF) [file pbio.0050229.sg003.pdf]

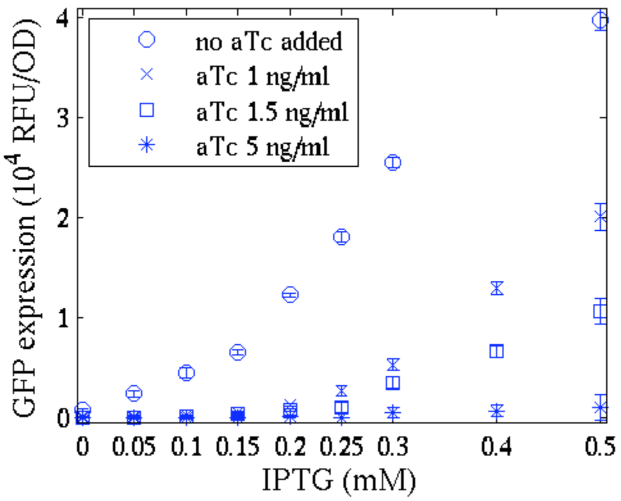

Figure S3. *Example for raw data, used to compile Fig. 2a of the main text.* "GFP expression" for strains (ZZS23) harboring $P_{Ltet\text{-}O1}$:*ryhB* on a plasmid, in addition to the $P_{Llac\text{-}O1}$:*crsodB-gfp* reporter. The IPTG dependence of GFP expression (defined from plots such as Figure S2) is plotted for different degrees of RyhB expression. The latter is controlled by the level of the inducer aTc in the growth medium as indicated by the legend.
