# Supplementary material for: Quantitative Characteristics of Gene Regulation by Small RNA
Source: PLoS Biol. 2007 Aug 21;5(9):e229. doi: 10.1371/journal.pbio.0050229 (PMC1994261; doi:10.1371/journal.pbio.0050229)
Supplement: Figure S4 — The fluorescence levels of cells carrying a plasmid coding for the target, PLlac−O1:crsodB-gfp, was measured as in Figure 2A, for strains ZZS21 (no ryhB) and ZZS23 (plasmid-borne ryhB). The fold of repression (vertical axis) is defined as the ratio between the two. The repression effect of RyhB is diminished at higher levels of IPTG, corresponding to higher transcription rates of the target. (45 KB PDF) [file pbio.0050229.sg004.pdf]

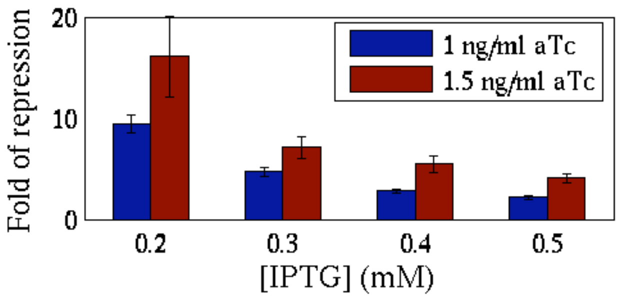


**Figure S4.** *Repression strength of RyhB depends on the transcription rate of the target*. The fluorescence levels of cells carrying a plasmid coding for the target, $P_{Llac-O1}$: *crsodB-gfp*, was measured as in Fig. 2a, for strains ZZS21 (no *ryhB*) and ZZS23 (plasmid-borne *ryhB*). The fold of repression (vertical axis) is defined as the ratio between the two. The repression effect of RyhB is diminished at higher levels of IPTG, corresponding to higher transcription rates of the target.
