# Supplementary material for: Quantitative Characteristics of Gene Regulation by Small RNA
Source: PLoS Biol. 2007 Aug 21;5(9):e229. doi: 10.1371/journal.pbio.0050229 (PMC1994261; doi:10.1371/journal.pbio.0050229)
Supplement: Table S1 — See Material and Methods for a detailed description of the fitting procedure. (13 KB PDF) [file pbio.0050229.st001.pdf]

Fitted values for a_s	
Chr-*ryh*B	0.7 ± 0.17
PryhB-*ryh*B	1.3 ± 0.14
Ptet-*ryh*B , aTc 1 ng/ml	2.5 ± 0.13
Ptet-*ryh*B , aTc 1.5 ng/ml	3.7 ± 0.19
Ptet-*ryh*B , aTc 2 ng/ml	10.4 ± 3.4
Fitted value for a_λ	0.15 ± 0.03

Table S1. Best-fit parameters of the data in Fig. 2a to model (1), given in terms of 50% confidence interval. See Material & Methods for a detailed description of the fitting procedure.
