# Supplementary material for: Quantitative Characteristics of Gene Regulation by Small RNA
Source: PLoS Biol. 2007 Aug 21;5(9):e229. doi: 10.1371/journal.pbio.0050229 (PMC1994261; doi:10.1371/journal.pbio.0050229)
Supplement: Table S2 — See Material and Methods for a detailed description of the fitting procedure. (13 KB PDF) [file pbio.0050229.st002.pdf]

| Fitted values for $a_s$ | |
|---|---|
| Ptet-*is10ou*t , aTc 2 ng/ml | 1.52 ± 0.05 |
| Ptet-*is10ou*t , aTc 6 ng/ml | 2.06 ± 0.11 |
| Ptet-*is10ou*t , aTc 10 ng/ml | 2.85 ± 0.10 |
| Fitted value for $a_\lambda$ | 0.65 ± 0.07 |

**Table S2.** Best-fit parameters of the data in Fig. 3a to model (1), given in terms of 50% confidence interval. See Material & Methods for a detailed description of the fitting procedure.
