# Supplementary material for: Quantitative Characteristics of Gene Regulation by Small RNA
Source: PLoS Biol. 2007 Aug 21;5(9):e229. doi: 10.1371/journal.pbio.0050229 (PMC1994261; doi:10.1371/journal.pbio.0050229)
Supplement: Table S3 — (13 KB PDF) [file pbio.0050229.st003.pdf]

Target		small RNA source	background
ZZS	0 none<br>1 pZE12G $P_{Llac-O1}$-*gf*p<br>2 pZE12S $P_{Llac-O1}$-*crsodB-gf*p<br>3 pZE12IS $P_{Llac-O1}$-*cris10-gf*p<br>4 Φ(*crsodB-gfp*)	0 none<br>1 pZA31-*lu*c $P_{Ltet-O1}$–*lu*c<br>2 chromosomal *ryh*B<br>3 pZA31R $P_{Ltet-O1}$-*ryh*B<br>4 pZA30R P_{ryhB}-*ryh*B<br>5 pZA31O PLtet–O1 -*is10ou*t	q *hfq*$^-$<br>s *sodB*$^-$

Table S3. Naming scheme for strains used in this study.
