# Supplementary material for: Quantitative Characteristics of Gene Regulation by Small RNA
Source: PLoS Biol. 2007 Aug 21;5(9):e229. doi: 10.1371/journal.pbio.0050229 (PMC1994261; doi:10.1371/journal.pbio.0050229)
Supplement: Text S1 — (120 KB PDF) [file pbio.0050229.sd001.pdf]

Quantitative features of gene regulation by small RNA – Supporting Information

E Levine[1], **Z Zhang**[2], **T Kuhlman**[1], **and T Hwa**[1]

Center for Theoretical Biological Physics[1] and Division of Biological Sciences[2], University of California at San Diego

1. Small RNA and a single target

Our theoretical study of the sRNA mediated repression starts with a mass-action model for a minimal system, consisting of a small RNA (s) and a single species of mRNA target (m) :

$$\begin{aligned}
\frac{d}{dt}s &= \alpha_s - \beta_s s - (k_+ ms - k_- c) + (1-p)\beta_c c \\
\frac{d}{dt}m &= \alpha_m - \beta_m m - (k_+ ms - k_- c) \\
\frac{d}{dt}c &= (k_+ ms - k_- c) - \beta_c c \; .
\end{aligned} \tag{1}$$

Here c is the concentration of sRNA-mRNA complexes, and p is the probability that degradation of the mRNA in the complex is accompanied by degradation of the sRNA. The limit $p = 0$ describes a catalytic mode of action, while the case $p > 0$ allows for mutual degradation and a non-catalytic interaction, as is the case of some endogenous *E. coli* small RNAs such as RyhB [1].

Assuming fast equilibration of the complex‡, namely $dc/dt \simeq 0$, we eliminate c from these equations, yielding

$$\begin{aligned}
\frac{d}{dt}s &= \alpha_s - \beta_s s - pkms \\
\frac{d}{dt}m &= \alpha_m - \beta_m m - kms \; ,
\end{aligned} \tag{2}$$

with $k = \beta_c k_+/(k_- + \beta_c)$. The case $p = 1$, corresponding to sRNA degradation accompanying *every* target-binding event, is depicted in Fig. 1c of the main text. Under this assumption, values of the model parameters can be estimated from various experiments, as described in Materials & Methds and summarized in Table 1.

Repression of gene expression in some cases does not involve mutual degradation. Rather, the non-catalytic nature of the interaction stems from the stability of the sRNA-mRNA complex. This, for example, characterizes the kinetics of CopA and its target, RepA, of the plasmid R1 copy control system [2], as well as the kinetics of the IS10 transposition regulator,

‡ To study steady-state properties, as done is this paper, this assumption is not required.

RNA-OUT [3]. Within our model, the β_c-terms now correspond only to dilution by growth, and therefore $p = 1$. Setting $k_- + \beta_c \simeq \beta_c$ in the above expression for k yields $k \simeq k_+$.

The steady-state behavior of the mRNA-sRNA system is given by the steady-state concentration of the two RNA species, m^* and s^*. The steady-state protein synthesis rate is then given by $P = \gamma m^*$, where γ is the rate of translation initiation. Setting $\frac{dm}{dt} = \frac{ds}{dt} = 0$ in (2) and solving for the steady-state concentration m^* we get

$$m^* = \frac{1}{2\beta_m}\left[(\alpha_m - \alpha_s/p - \lambda) + \sqrt{(\alpha_m - \alpha_s/p - \lambda)^2 + 4\alpha_m\lambda}\right] , \qquad (3)$$

where

$$\lambda = \frac{\beta_m \beta_s}{k\,p}$$

is a 'leakage rate', characterizing the turnover of mRNA *not* due to interaction with the sRNA. In the limit of strong mRNA-sRNA interaction, $\lambda \ll 1$, this expression is simplified to a piece-wise linear form,

$$m^* = \begin{cases} \frac{\alpha_m - \alpha_s/p}{\beta_m} + \frac{\alpha_s/p}{\beta_m}\frac{\lambda}{\alpha_m - \alpha_s/p + \sqrt{\lambda}} & \text{for } \alpha_m \geq \alpha_s/p \ , \\ \frac{\alpha_m}{\beta_m}\frac{\lambda}{\alpha_s/p - \alpha_m + \sqrt{\lambda}} & \text{for } \alpha_m \leq \alpha_s/p \ . \end{cases} \qquad (4)$$

This expression reduces to the idealized picture of Fig. 1c as $\lambda \to 0$. Thus, the target gene is silenced for $\alpha_m < \alpha_s/p$, and is gradually activated as α_m is increased beyond this threshold. The effect of the sRNA becomes less substantial as α_m increases, and in the limit $\alpha_m \gg \alpha_s/p$ we have $m^* \simeq \alpha_m/\beta_m$, as if the small RNA does not exist. In Section 3 we discuss how this is very different from what happens with a protein repressor.

The onset of mRNA expression is given by $\alpha_m \simeq \alpha_s/p$, and hence can be controlled dynamically, namely through biochemical signals rather than through mutation. We note that this expression threshold is hardly dependent on the strength of the sRNA-mRNA interaction itself, as long as the interaction is sufficiently strong. What does depend on the interaction strength is the abruptness of the transition, or its "sensitivity", customarily quantified through the slope of the response curve $m^*(\alpha_s)$ in a double-log scale, $\max \frac{d\log m^*}{d\log \alpha_s}$. According to our model, it is given by $\frac{1}{2}\sqrt{pk\alpha_m/\beta_m\beta_s} = \frac{1}{2}\sqrt{\alpha_m/\lambda}$. With the parameters of Table 1 we find the maximum sensitiy to be given approximately by 2.5 for $\alpha_s = 1$ nM/sec, and 4.3 for $\alpha_s = 3$ nM/sec. In comparison, for a protein repressor the sensitivity is bounded by the Hill coefficient, and is typically $\lesssim 2$. An alternative characterization of sensitivity, the response coefficient [4, 5], can be shown to take values ≥ 9, corresponding to Hill coefficient ≤ 2.

2. Small RNA with multiple targets

Most of the known small RNAs have multiple targets. The level of complementarity between the small RNA and its binding site on the mRNA molecule may be different from target to target. This variability in sequence is expected to lead to a variability in the binding strength, although no data is available to quantify this effect. It is straightforward to incorporate the multiplicity of targets into our model. For example, for two target mRNAs, m_R and m_T, one

has §||

$$
\begin{aligned}
\frac{d}{dt}s \quad &= \alpha_s - \beta_s s - (k_R m_R + k_T m_T)\, s \\
\frac{d}{dt}m_R &= \alpha_R - \beta_R m_R - k_R m_R s \\
\frac{d}{dt}m_T &= \alpha_T - \beta_T m_T - k_T m_T s\ .
\end{aligned} \tag{5}
$$

We assume that the two genes are expressed from different promoters, and can thus be controlled by different signals. We therefore allow for α_R and α_T to change independently.

Consider an experiment in which the activity of the reporter gene (*geneR*) $\alpha_R < \alpha_s$ is held fixed, while the activity α_T of another target gene (*geneT*) is increased (say, by an external stimulus; see Fig. S1). This thought-experiment is a simplified version of the experiment presented in Fig. 4b. In that case, *geneR* is either chromosomal *sodB* or chromosomal *fumA*, while *geneT* is the plasmid-borne target *crsodB-gfp*. The effect of α_T and k_T on the expression level of *geneR* is depicted in Fig. 4c of the main text. To draw this figure we used an explicit solution of Eq. 5, with $\beta_T = \beta_R$ and $\alpha_s = 5\alpha_R$. As in the case of a single target, this solution depends on the values of β_R, β_T and k_R, k_T only through $\lambda_R = \beta_R\beta_s/k_R$, $\lambda_T = \beta_T\beta_s/k_T$.

In the strong interaction limit, $\lambda \ll 1$, we find

$$
\begin{aligned}
&\text{For } (\alpha_R + \alpha_T) \gg \alpha_s\,, \quad m_R^* = \tfrac{\alpha_R}{\beta_R}\left(1 + \tfrac{\lambda_T}{\lambda_R}\tfrac{\alpha_s}{\alpha_T}\right)^{-1} \text{ and } m_T^* = (\alpha_T - \alpha_s)/\beta_T\,; \\
&\text{For } (\alpha_R + \alpha_T) < \alpha_s\,, \quad m_R^* = m_T^* = 0\,.
\end{aligned} \tag{6}
$$

With α_R fixed, one can think of the threshold for gene expression as being set by $\alpha_R + \alpha_s$. As long as α_T is below this threshold, both genes are silenced. Once α_T exceeds the threshold, mRNA molecules of both species survive the sRNA repression, and both genes start being expressed. When *geneT* is highly expressed, its expression grows proportionally to α_T, with the usual shift due to the sRNA effect. In this limit the expression of *geneT* is not affected by the presence of *geneR*. At the same time, the expression of *geneT* is relieved from the sRNA effect ($m_R \simeq \alpha_R/\beta_R$), an effect that is amplified if $\lambda_T \gg \lambda_R$. This can be easily explained, as high abundance of m_R, and/or strong interaction with the sRNA, directs all sRNAs to annihilate mRNA molecules of *geneT* rather than of *geneR*. Indirectly, *geneT* acts as an activator of *geneR*.

3. Protein repressor

In this Section we compare the features of sRNA mediated repression with those of a protein repressor. The effect of a transcription factor, which represses transcription initiation by blocking binding of the RNA polymerase has been shown [6] to take the form

$$
m^* = \frac{\alpha_m}{\beta_m}\frac{1}{1 + (r/K)^n}\ . \tag{7}
$$

§ For simplicity we set hereafter $p = 1$.

|| More generally, our model is given by $\frac{d}{dt}s = \alpha_s - \beta_s s - (\sum_i k_i m_i)\, s$ and $\frac{d}{dt}m_i = \alpha_i - \beta_i m_i - k_i m_i s$, where i enumerates the different targets.

Here r is the concentration of the protein repressor, K its binding affinity to the promoter, and n is the Hill coefficient which describes the effect of cooperativity (typically one has $n \lesssim 2$). The effect of the protein repressor is multiplicative. The fold of repression is determined solely by the properties of the repressor (its binding affinity to the promoter, its concentration, etc).

Proteins can also take part in post-transcriptional regulation. A protein regulator can act by destabilizing a target mRNA, either directly (*e.g.* by recruiting a ribonuclease) or indirectly (by blocking translation). For example, the protein CsrA inhibits translation of the glgCAP mRNA by binding to its 5'-UTR [7], thus exposing it to degradation. As a crucial difference from a small RNA, the protein is assumed to be unaffected by the degradation of the RNA molecule, and would be recycled to exert its effect on other molecules. In terms of mass-action equations, this can be modeled by

$$\begin{aligned} \frac{d}{dt}R &= \alpha_R - \beta_R R - k_{+1} m R + k_{-1} C + \beta_C C \\ \frac{d}{dt}m &= \alpha_m - \beta_m m - k_{+1} m R + k_{-1} C \\ \frac{d}{dt}C &= k_{+1} m R - k_{-1} C - \beta_C C \ . \end{aligned} \tag{8}$$

Here C is the concentration of mRNA:protein complex, k_{+1} and k_{-1} are the binding and unbinding rates of this complex, and β_c is the degradation rate of the mRNA when it is attached to the protein. α_R and β_R are, respectively, the production and decay rates of the protein.

Perhaps surprisingly, the solution of model (8) suggests that the steady state effect of this mode of repression is similar to that of a transcriptional repressor. In the case where the protein repressor inhibits translation, we have

$$\text{Protein production} \sim m^* = \frac{\alpha_m}{\beta_m} \frac{1}{1 + \frac{\beta_c}{\beta_m} \frac{r}{K}} \ , \tag{9}$$

with $K^{-1} = k_{+1}/(k_{-1} + \beta_c)$. If the repressor does not interfere with translation, all mRNA are actively translated, and one has

$$\text{Protein production} \sim m^* + C^* = \frac{\alpha_m}{\beta_m} \frac{1 + \frac{r}{K}}{1 + \frac{\beta_c}{\beta_m} \frac{r}{K}} \ . \tag{10}$$

More generally, it is possible that multiple proteins can bind cooperatively to the mRNA molecule to inhibit its translation or promote its degradation (as is the case of CsrA [7]). It is simple to show that this would result in a Hill coefficient > 1, as in (7).

In common to all the cases considered, the repressor reduces the abundance of mRNA by a constant multiplicative factor ("fold-change"), irrespective of the activity of the promoter (Fig. 5b of the main text). In other words, the effect of an enzymatic regulator is multiplicative, whereas the effect of sRNA is additive. However, if a certain target is always expressed less efficiently than its sRNA regulator ($\alpha_m \ll \alpha_s$), the effect of the latter becomes multiplicative, as can be seen from (4). This is due to the fact that a weak target cannot change significantly the level of sRNA.

4. Noise in the "silenced" state

In the repressed state, the average number of mRNA molecules in the cell, and with them the number of proteins, are kept very low. In such a state, fluctuations in the copy number may become significant. Here we compare the noise properties in the repressed state of two systems: sRNA-mediated mRNA destabilization, and transcription repression.

The nature of leakage is different in the two systems. For the system regulated by sRNA, many short-lived transcripts are produced. The size b of protein 'bursts', namely the number of proteins translated from a single mRNA molecule, is small. While the large number of transcript events would make it improbable to have no proteins at all, the small size of the bursts would also make it unlikely to have any large amount of proteins. Conversely, for the system regulated at transcriptional level, the number of transcription events is kept low. However, any transcription event can result in a relatively large burst of proteins, and thus it is plausible that the cell would be overpopulated by proteins for short periods of time.

In Fig. 5c of the main text we present results of Monte-Carlo simulations [8] for two genes. *geneA* represents a mRNA targeted by a small regulatory RNA, and *geneP* represents a gene repressed at the stage of transcription. Transcriptional repression is mimicked by choosing the transcription rate of *geneP* to be such that the average copy number of mRNA molecules (and thus, of proteins) would be the same for the two genes. For the parameters used in our simulations with $\alpha_s = 1\,\mathrm{nM}/\min, k_A = 0.02(\mathrm{nM}\cdot\min)^{-1}$, and $\alpha_A = 0.1\,\mathrm{nM}/\min$ for *geneA*, this corresponds to $\alpha_P = 0.0043\,\mathrm{nM}/\min$ for *geneP*. Other parameters are as given in Table 1. Data were collected at $t \simeq 1000\ \mathrm{min}$ from 10^4 independent simulations.

Two major features arise from Fig 5c. First, we find that the number of proteins/cell is much more peaked around the mean value (~ 2.15), i.e. the variance is much lower, for the sRNA regulated gene. Second, we find that the probability of having no proteins at all is much higher in the case of transcription repression.

To get better understanding of these features, we assume a high level of sRNA (as expected in the repressed state), and thus neglect fluctuations in its copy number. We therefore take $s = (\alpha_s - \alpha_m)/\beta_s$. With this assumption, one can think of the target mRNA as having an effective degradation rate $\tilde{\beta}_m = \beta_m + k(\alpha_s - \alpha_m)/\beta_s$. Following [9], we expect

$$\langle p \rangle = \frac{\alpha_m}{\tilde{\beta}_m}\frac{\alpha_p}{\beta_p} \tag{11}$$

$$\frac{\langle \Delta p^2 \rangle}{\langle p \rangle} = 1 + \frac{\alpha_p/\tilde{\beta}_m}{1+\beta_p/\tilde{\beta}_m} \simeq 1 + \alpha_p/\tilde{\beta}_m\ , \tag{12}$$

where $\langle p \rangle$ is the mean protein number, and $\langle \Delta p^2 \rangle$ is its variance. The expression in (12) is the Fano factor, a common measure for the magnitude of the noise. Importantly, we see that it is linear in the mRNA life-time, which is substantially reduced by the small RNA. Moreover, we note that the Fano factor is independent of the transcription rate α_m.

References

[1] Masse E., Escorcia F.E. & Gottesman S. Coupled degradation of a small regulatory RNA and its mRNA targets in Escherichia coli. Genes. Dev. 17, 2374-2383 (2003).

[2] Hjalt TA & Wagner EG Bulged-out nucleotides in an antisense RNA are required for rapid target RNA binding in vitro and inhibition in vivo. Nucleic Acids Res 23, 580-587 (1995).

[3] Case CC, Simons EL, Simons RW. The IS10 transposase mRNA is destabilized during antisense RNA control. Embo J 9, 1259-1266 (1990).

[4] Koshland, D. E., Jr., Nemethy, G. & Filmer, D. Biochemistry 5, 365-385 (1966).

[5] Goldbeter, A, & Koshland DE, J. Proc Natl Acad Sci USA, 78, 6840-4 (1981).

[6] Bintu L., Buchler N.E., Garcia H.G., Gerland U., Hwa T., Kondev J. & Phillips R. Transcriptional regulation by the numbers: models. Curr. Op. Genet. Dev. 15, 116-124 (2005); Bintu L., Buchler N.E., Garcia H.G., Gerland U., Hwa T., Kondev J., Kuhlman T. & Phillips R. Transcriptional regulation by the numbers: applications. Curr. Op. Genet. Dev. 15, 125-135 (2005).

[7] Baker C.S., Morozov I., Suzuki K., Romeo T. & Babitzke P. CsrA regulates glycogen biosynthesis by preventing translation of glgC in Escherichia coli. Mol Microbiol. 44, 1599-610 (2002).

[8] Gillespie D.T. Exact stochastic simulation of coupled chemical reactions. J. Phys. Chem 81, 2340-61 (1977).

[9] Thattai M. & van Oudenaarden A.. Intrinsic noise in gene regulatory networks. Proc Natl Acad Sci USA. 98(15), 8614-9 (2001).
